# Nasal-Type Natural Killer/T-cell Lymphoma With Inaugural Testicular Presentation in a Young Patient

**DOI:** 10.7759/cureus.54733

**Published:** 2024-02-22

**Authors:** Oumayma Mnafe, Eddou Hicham

**Affiliations:** 1 Hematology and Medical Oncology, Military Hospital Moulay Ismail Meknes, Meknes, MAR

**Keywords:** nk t cell lymphoma, pink score, bilateral adrenal masses, testicular mass, mogad

## Abstract

Nasal-type natural killer/T-cell lymphoma is a rare non-Hodgkin's malignant lymphoma. A distinct clinicopathological entity associated with the Epstein-Barr virus, it typically presents with otorhinolaryngeal symptomatology.

We report a rare case of a 24-year-old patient with nasal-type lymphoma with an atypical inaugural testicular presentation associated with the discovery of bilateral adrenal involvement.

## Introduction

Nasal-type natural killer/T-cell (NK/T) lymphoma is a non-Hodgkin's lymphoma. This aggressive nasal-type malignant lymphoma, previously known as lethal midline granuloma, is a distinct clinicopathological entity associated with the Epstein-Barr virus (EBV) that typically causes destruction of the medial, palatal, and orbital walls [1].

It is a very rare lymphoma in Africa and Europe but much more frequent in regions where EBV is endemic, such as East Asia, South-East Asia, and Central and South America [2].

It is a clinical entity involving necrosis that begins preferentially in the nasal cavities and nasopharynx and extends to the centrofacial bony structures, with a spontaneous fatal course. Its clinical presentation is mainly otorhinolaryngeal (ORL) symptomatology, with a median age of diagnosis of 50 years.

We report a rare case of a 24-year-old patient with nasal-type NK/T lymphoma with an atypical inaugural testicular presentation.

## Case presentation

**Figure 1:**
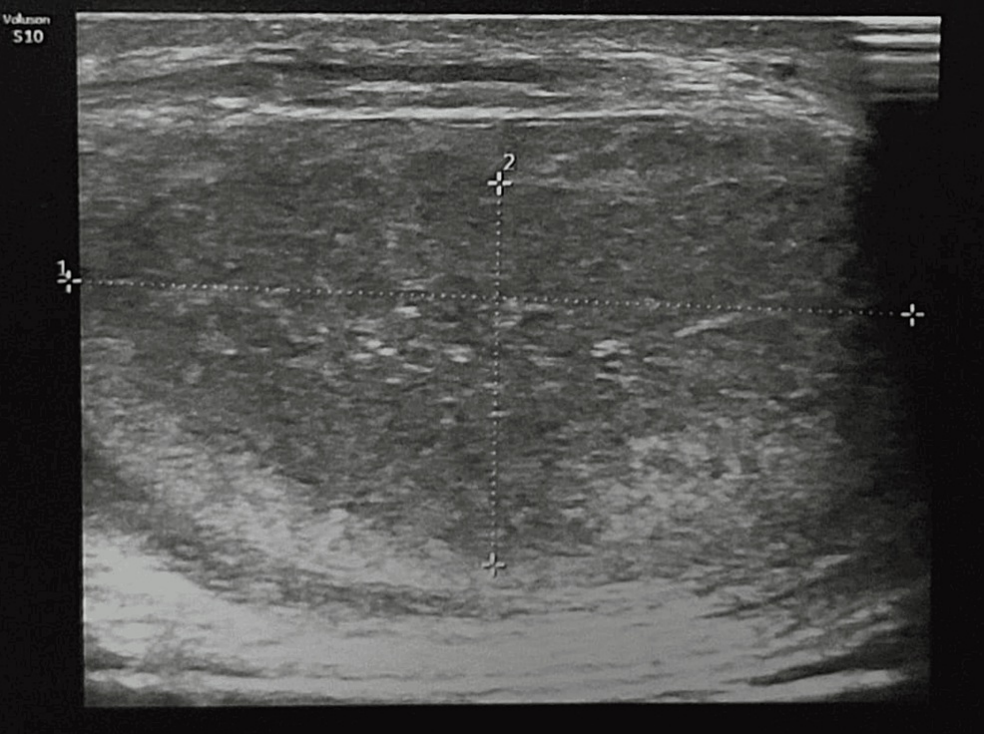
Patient's scrotal ultrasound A left scrotal ultrasound suggested a suspicious-looking mass.

A 24-year-old patient with no previous history presented to the urology department for the management of a large, painless bursa on the left side, with no inflammatory signs. A scrotal ultrasound revealed a suspicious mass (Figure 1). An orchiectomy was performed, with an anatomopathological study of the lesion, but this was inconclusive.

A cervico-thoraco-abdomino-pelvic (CTAP) CT scan performed as part of the extension work-up revealed two bilateral adrenal masses. An adrenal biopsy was then performed, which came back in favor of a lymphoma.

The history revealed the notion of intermittent nasal obstruction with B signs of weight loss in less than one month. The patient's Eastern Cooperative Oncology Group (ECOG) performance status was 2 and the clinical examination revealed no tumor syndrome.

Adrenal and nasal biopsies showed a lymphoid proliferation of pleomorphic atypical cells with regular nuclei, with angioinvasion images of perivascular arrangement associated with areas of ischemic necrosis. Immunohistochemistry showed diffuse, intense staining for CD3+ and CD56+, with no staining for CD4, CD7, CD5, CD30, CD8, epithelial membrane antigen (EMA), or EBV. Fluorescence in situ hybridization (FISH) testing for EBV using an Epstein-Barr virus-encoded small RNA (EBER) probe was not performed. Ki67 was estimated at 50%.

On the basis of this anatomical and clinical comparison, the diagnosis of nasal-type NK/T lymphoma with testicular and adrenal localization was accepted.

The blood count showed no lymphocytosis or cytopenias. Lactate dehydrogenase (LDH) levels were elevated. Cortisolaemia was depressed. EBV polymerase chain reaction (PCR) was not performed.

**Figure 2:**
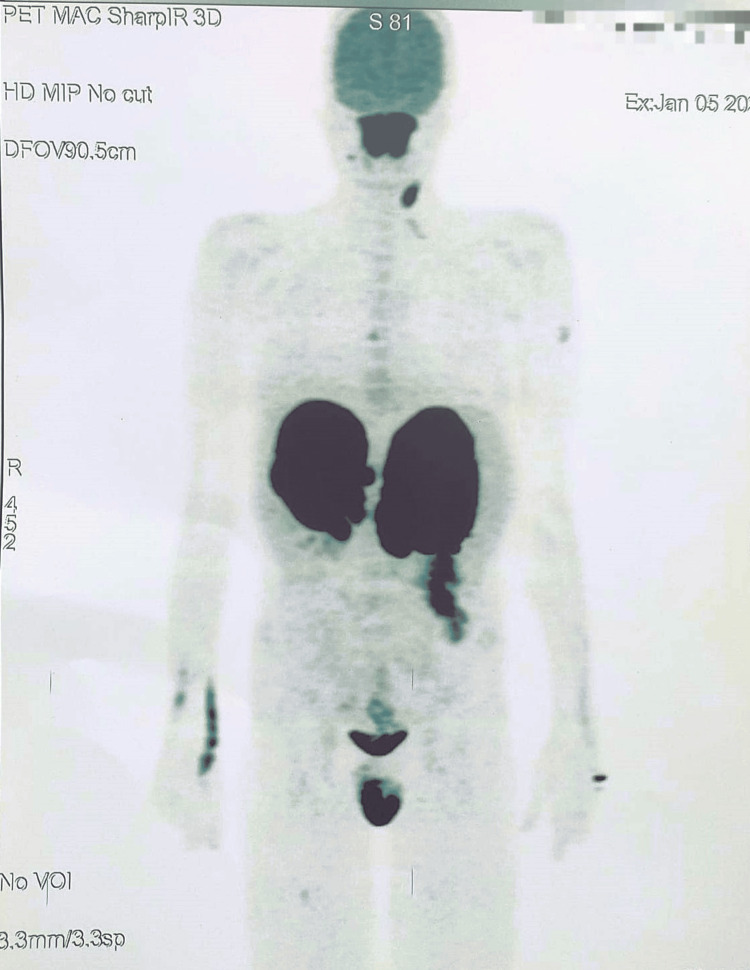
PET scanner Hypermetabolic thickening of nasal, maxillary, and ethmoidal sinuses, associated with active foci of bilateral cervical lymph nodes. Hypermetabolic tumor masses of the adrenal glands with necrotic centers: right SUV = 13.2 of 100*117*156 mm; left SUV = 14.7 of 127*95*172 mm. Very intense diffuse pathological testicular hypermetabolism (SUV = 11.7). Diffuse pathological hypermetabolism of the upper left colon (SUV = 8). Hepatic SUV: 0.9. SUV: standardized uptake value.

A lumbar puncture was performed to rule out CNS involvement, and a fluorodeoxyglucose (FDG) PET scan revealed bilateral adrenal involvement (maximum standardized uptake value (SUVmax) of 13.2 on the right and 14.7 on the left), testicular involvement (SUVmax = 11.7), and left colon involvement (SUVmax = 8), with pathological thickening of the maxillary and ethmoidal nasal sinuses (SUVmax = 12.7) ( Figure 2).

The NK/T lymphoma was stage IV according to the Ann Arbor classification, and high risk according to the Prognostic Index of Natural Killer Cell Lymphoma (PINK) score, depending on age, stage, presence of extra-nasal involvement, and presence of distant adenopathies.

The therapeutic decision was chemotherapy followed by autologous stem cell transplantation if a complete response was achieved. The proposed chemotherapy was the MOGAD protocol consisting of a combination of methotrexate, oxaliplatin, gemcitabine, asparaginase, and dexamethasone.

PET-CT re-evaluation after four courses showed a complete metabolic response (Deauville score 1), and an autologous stem cell transplantation was performed without incident. Progression at one year was satisfactory; the patient presented no tumor or ORL symptoms. The patient would benefit from clinical-biological follow-up and PET-CT imaging to ensure maintenance of the complete response and early detection of any possible relapse.

## Discussion

Nasal-type NK/T lymphoma is a lymphoma developed at the expense of cytotoxic cells: natural killer (NK) cells or T cells. NK cells belong to the innate immune system. Unlike T lymphocytes, they do not require specific immunization to exert their cytotoxic activity [3]. They play an essential role in the regulation of viral infections. The cytotoxic effect of NK cells is mainly due to the release of proteins such as granzymes and perforin, contained in their intracytoplasmic granules [3]. This type of lymphoma is a rare clinical and anatomopathological entity. The pathogenesis of this entity is unknown but involves infection with EBV, which is associated with a poor prognosis [4].

The median age at diagnosis is 45 to 50 years old, with a male predisposition (approximately two men for one woman) [3]. Clinical presentation is highly heterogeneous. Typically, the main presenting features are involvement of the nasal cavity and upper aero-digestive tract, but extra-otorhinolaryngeal involvement is possible in 20-40% of patients (extra-nasal form). Positive diagnosis of nasal-type NK/T lymphoma is based on histopathological examination and molecular biology of lesional tissue [1]. However, precise histopathological interpretation is often difficult due to the friable nature of the tissue and its often necrotic nature. Indeed, necrosis may necessitate repeated biopsies of lesional and peri-lesional tissue [1]. In the case of extra-nasal presentation of NK/T lymphoma, a systematic search should therefore be made for nasal involvement, which may be minimally present [3].

**Figure 3:**
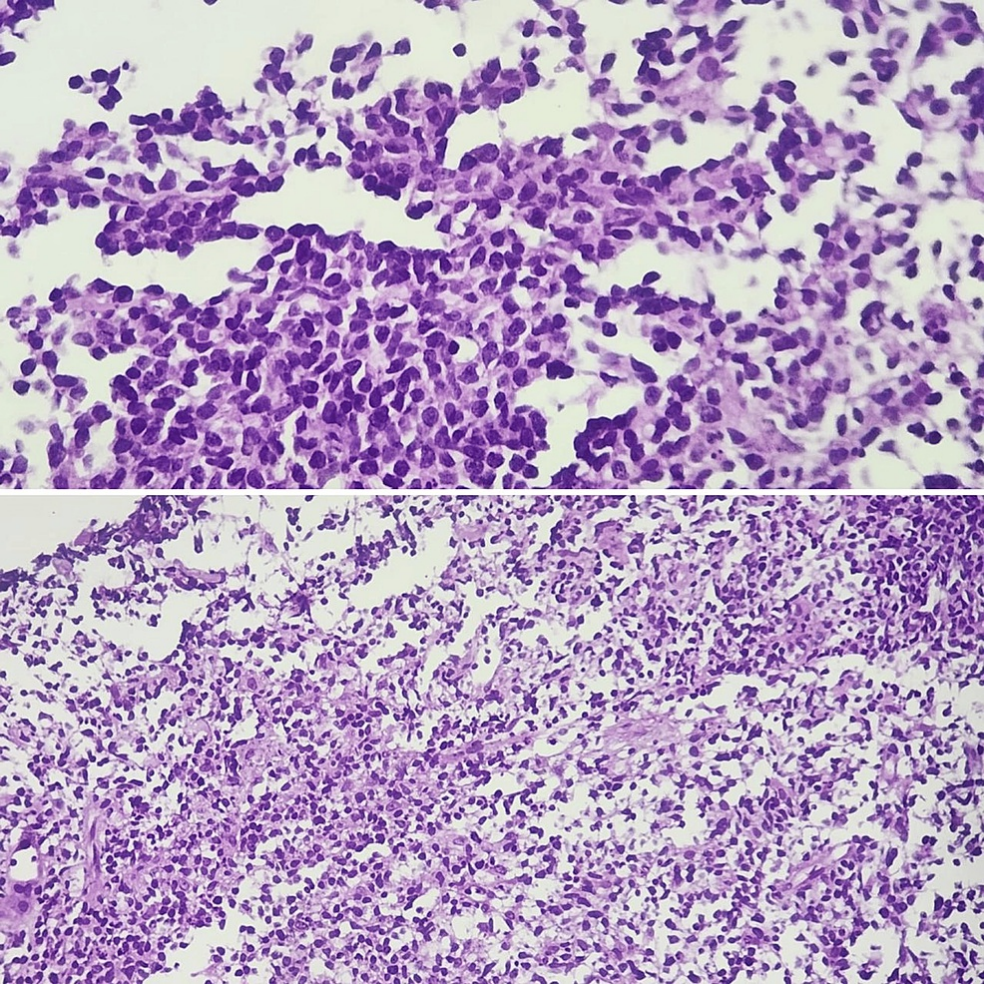
Histological aspects of adrenal and testicular biopsies Pleomorphic atypical cells with regular nuclei, with angioinvasion images of perivascular arrangement associated with areas of ischemic necrosis.

Histological examination reveals sheets of atypical cells that may be small, medium, large, or sternbergoid giants [4]. Characteristic of nasal NK/T lymphoma is the presence of vascular lesions, with tumor cells arranged in perivascular sleeves (angio-centrism) and penetrating the vascular wall, occupying the lumen and forming vascular thrombi (angio-destructive lesions) (Figure 3) [4,5]. Immunophenotypic studies reveal the expression of cellular markers of T lymphocytes and NK lymphocytes, hence the name of this lymphoma. The immunophenotype most typical of nasal-type NK/T lymphoma is CD2+, CD56+, which is the specific marker of NK cells, with expression of intracytoplasmic anti-CD3 antibody with surface CD3 negativity [4,6].

The initial work-up for NK/T lymphoma extension relies on PET-CT to characterize and detect extra-nodal lesions, and to stage the lymphoma according to the Ann Arbor classification. It is also essential to perform a blood EBV PCR and monitor it during treatment. A high EBV viral load is associated with extensive disease, unfavorable response to treatment, and poorer survival [3]. Some studies also suggest the use of EBV PCR as an indicator of minimal residual disease, especially in the asparaginase era [2]. Indeed, EBV-DNA positivity after treatment may predict early relapse and a poor prognosis for patients with early-stage NK/T lymphoma [2].

A cytogenetic study has concluded the presence of a variety of cytogenetic aberrations such as mutation of the Fas suppressor gene and mutation of the P53 suppressor gene. However, the most common cytogenetic abnormality is a deletion on chromosome 6 in the q21 q25 region or the p10 region [4].

The reference prognostic score is the PINK score, based on age over 60 years old, diffuse stage, presence of distant adenopathy, and extra-nasal involvement [5]. EBV viral load can also be included in the calculation of the PINK score, known as the PINK-E score [5]. The PINK-E score can be used to define three risk groups: the low-risk group, the intermediate-risk group, and the high-risk group, as in our patient.

Recently, a new prognostic model, PINK-B, has been proposed to predict the clinical outcome of patients treated with non-anthracycline-based strategies [5]. Elevated serum beta-2 microglobulin (B2M) has been suggested as a potential prognostic predictor for patients, under the PINK-B score.

NK/T lymphoma is an aggressive lymphoma with a generally poor prognosis [3]. The five-year survival rate for localized forms (Ann Arbor stages I and II) is around 75% [6,7], and for disseminated forms (Ann Arbor stages III and IV) around 47% [8].

Treatment of nasal-type NK/T lymphoma is currently based on chemotherapy containing asparaginase and gemcitabine using MOGAD or SMILE-type protocols, followed by intensive autograft or allograft of stem cells treatment. It is also important to recognize the role of radiotherapy in the management of nasal-type NK/T lymphoma. The initial response to radiotherapy is so rapid and dramatic that the use of involved-field radiotherapy has been accepted as the preferred treatment option for localized, stage I, and stage II forms [9].

Radiotherapy appears to have the greatest benefit if administered early and when the disease is localized, whereas it has a limited benefit when administered as salvage therapy to patients who do not achieve a complete response with chemotherapy [10].

## Conclusions

Although the classic presentation of NK/T lymphomas is otorhinolaryngeal symptomatology, it can also have an atypical initial presentation, testicular and adrenal, as in our patient. Precise histopathological interpretation is often difficult, making anatomopathological and clinical expertise essential for diagnosis.

NK/T lymphoma is an aggressive lymphoma with an unfavorable prognosis overall. The therapeutic decision must be taken by a multidisciplinary consultation.
